# How to Integrate Prostate Cancer Biomarkers in Urology Clinical Practice: An Update

**DOI:** 10.3390/cancers16020316

**Published:** 2024-01-11

**Authors:** Catalin Baston, Adrian Preda, Alexandru Iordache, Vlad Olaru, Cristian Surcel, Ioanel Sinescu, Constantin Gingu

**Affiliations:** 1 Department of Nephrology, Urology, Immunology and Immunology of Transplant, Dermatology, Allergology, Faculty of Medicine, “Carol Davila” University of Medicine and Pharmacy, 050474 Bucharest, Romania; catalin.baston@umfcd.ro (C.B.); vlad.olaru@umfcd.ro (V.O.); cristian.surcel@umfcd.ro (C.S.); ioanel.sinescu@umfcd.ro (I.S.); constantin.gingu@umfcd.ro (C.G.); 2 Center of Uronephrology and Kidney Transplantation, Fundeni Clinical Institute, 258 Fundeni Street, 022328 Bucharest, Romania; iordachemd@gmail.com

**Keywords:** biomarkers, prostate cancer genetics, clinical practice, personalized medicine, individualized medicine

## Abstract

**Simple Summary:**

The management of prostate cancer becomes more and more challenging mainly due to limitations of risk stratifications systems and its heterogenous clinical and biological behavior. Therefore, to increase detection rate of clinically significant prostate cancer and to assess the aggressiveness of the disease is a clinical priority in daily urology practice. Several biomarkers have been developed over the last decade to improve the accuracy of serum PSA and to help clinicians in difficult management scenarios. In this review article, we focus on the scientific evidence that supports the clinical use of the most robust and reliable biomarkers considered by professional urological societies (and included in uro-oncological guidelines) in the early diagnosis process and treatment decisions for clinically localized or advanced prostate cancer.

**Abstract:**

Nowadays, the management of prostate cancer has become more and more challenging due to the increasing number of available treatment options, therapeutic agents, and our understanding of its carcinogenesis and disease progression. Moreover, currently available risk stratification systems used to facilitate clinical decision-making have limitations, particularly in providing a personalized and patient-centered management strategy. Although prognosis and prostate cancer-specific survival have improved in recent years, the heterogenous behavior of the disease among patients included in the same risk prognostic group negatively impacts not only our clinical decision-making but also oncological outcomes, irrespective of the treatment strategy. Several biomarkers, along with available tests, have been developed to help clinicians in difficult decision-making scenarios and guide management strategies. In this review article, we focus on the scientific evidence that supports the clinical use of several biomarkers considered by professional urological societies (and included in uro-oncological guidelines) in the diagnosis process and specific difficult management strategies for clinically localized or advanced prostate cancer.

## 1. Introduction

In urology clinical practice, prostate cancer (PC) is the most commonly diagnosed malignancy and the leading cause of cancer-related death in this population [1]. Moreover, PC is a major public health issue worldwide despite continued efforts to improve its prognosis and clinically relevant outcomes (cancer-specific survival, overall survival) following the introduction of an increased number of available treatment options and approved therapeutic agents [1].

The discovery of serum PSA has revolutionized the diagnostic process and disease management of PC. Up to the present time, due to the best scientific evidence, PSA is the only and universally accepted biomarker in urological practice worldwide for early diagnosis (screening), definitive diagnosis (prostate biopsy), prognosis, and therapeutic decisions. However, the serum PSA test showed modest performance characteristics in detecting any PC or clinically significant PC (csPC), defined by Gleason score ≥ 7, in both historical and contemporary cohorts. In a systematic review, a PSA cutoff level of 4 ng/mL had a sensitivity of 21% for the detection of any PC and a sensitivity of 51% for the detection of csPC [2]. Serum PSA is not a cancer-specific biomarker, and routine population-based screening using PSA testing was frequently associated with clinical harm (increased risk of overdiagnosis and overtreatment) [3]. In addition, there is no universally accepted serum PSA value to accurately indicate a prostate biopsy. Moreover, serum PSA cannot differentiate between indolent (who will never progress and consequently will not need any treatment) and aggressive forms (mostly likely to progress and associated with fatal outcomes) of PC. Therefore, urological associations and experts recommend nowadays the use of serum PSA together with other tools for screening purposes and to assess the opportunity of prostate biopsy or identify the aggressive behavior of PC.

Numerous biomarkers and prognostic/predictive tools have been developed to improve the accuracy of serum PSA in the diagnosis process, prognosis, and response to treatment. Emphasis was placed on the ability to identify csPC and to help clinicians in difficult-to-make-decisions situations such as prebiopsy (to biopsy or not a biopsy), pre-treatment (identification of a subgroup of patients who will most benefit from active surveillance (AS), specific curative therapies, or a multimodal approach), or post-treatment (helping to choose the most beneficial adjuvant treatment).

Yet, there is an ongoing concern among urologists and a stressful challenge in managing PC since the disease has a recognized heterogenous clinical and biological behavior among patients included in the same prognostic-risk group. In our daily uro-oncological practice, we must manage PC’ patients with either localized, advanced, or progressive disease but with indolent or aggressive behavior of the disease, irrespective of the initial disease stage or treatment strategy. To date, despite improvements in PC staging, imaging, pathology, and risk stratification tools, there are no means to accurately differentiate indolent from aggressive forms of PC or to predict the aggressiveness or responsiveness of a specific therapy. Adequate characterization of each PC patient from the initial diagnosis but also during the disease course is crucial for establishing an appropriate therapeutic strategy and improving specific survival.

In view of advances in our understanding of PC biology (carcinogenesis and disease progression), and more difficult management of this disease due to the increased number of treatment modalities and continuous approval of new therapeutic agents, the aim of this article is to review the data and scientific evidence that support the clinical utility of several biomarkers in the diagnosis and treatment strategy of PC.

## 2. Biomarkers for Initial Diagnosis Process (Prebiopsy)

Nowadays, due to the reported increasing incidence of PC and metastatic forms among patients diagnosed following the 2012 USPSTF (The U.S. Preventive Services Task Force) recommendation [4], to optimize the initial diagnosis process with the aim of increasing the detection rate of csPC and to reduce the potential harms related to biopsy procedures (overdiagnosis or overtreatment), many urological associations strongly recommend the use of serum PSA together with other biomarkers, magnetic resonance imaging (MRI), or tools such as risk calculators (RC) in a shared decision-making process. In 2021, the EAU (European Association of Urology) developed a risk-adapted strategy for early PC detection based on an initial serum PSA value in properly counselled patients aged 50 to 70 yrs followed by a step-by-step post-screening risk stratification algorithm for patients with PSA value ≥ 3 ng/mL using RC based on family history, PSA density, PSA velocity, digital rectal examinations (DRE), and subsequently MRI nomograms to identify high-risk patients harboring csPC at systematic ± targeted biopsy. In the authors’ opinion, this strategy will have a positive impact on reducing PC-specific mortality rates and the incidence of advanced-stage disease and will avoid the overdiagnosis/overtreatment associated risks [5]. ERSPC-RC (the European Randomized Study of Screening for Prostate Cancer risk calculator) and PCPT-RC (the Prostate Cancer Prevention Trial Risk Calculator) are examples of the two of the most widely used RC developed and independently validated to estimate the risk of PC detection and high-grade PC (HGPC) by performing prostate biopsy. Noteworthy, the diagnostic accuracy of these RCs can be improved by the inclusion of a serum PHI (Prostate Health Index) score or urinary biomarkers (PCA3-Prostate Cancer Antigen 3, MiPS-Michigan Prostate Score, T2: ERG), particularly for the detection of csPC. 

To help urologists and their patients in the initial diagnosis process of PC, particularly in the decision to perform a prostate biopsy or re-biopsy, to avoid unnecessary biopsies, to increase the specificity without missing a substantial number of csPC, but also to limit the risks associated with prostate biopsies (overdiagnosis/overtreatment), mainly for serum PSA in the gray zone, several serums, and urinary and tissue biomarkers have been developed (Table 1 and Table 2) [6,7]. 

Although there are many issues regarding the clinical utility and cost-effectiveness of these biomarkers (insufficient and inconsistent results, numerous retrospective and underpowered studies, lack of clinical validation and comparative studies, no optimal cutoff test value established), current uro-oncological guidelines for early detection of PC recommend considering such additional testing before making the decision to perform a biopsy. In the present context of our efforts to change PC screening strategy by increasing detection of csPC with missing as few cases as possible and significant clinical benefit and widespread use provided by multiparametric (mp) MRI in the initial diagnosis process of PC, it is important to reevaluate the role of these biomarkers and to establish which patients are most likely to benefit from MRI or biomarker use, in which diagnostic setting and sequence.

### 2.1. Serum Biomarkers

#### 2.1.1. Prostate Health Index (PHI) (Beckman Coulter Inc., Brea, CA, USA)

PHI is an FDA-approved test that uses the serum values of free PSA, [-2] proPSA, and total PSA to calculate a prognostic score (PHI score) according to the formula ([-2] proPSA/free PSA) × √total serum PSA for detecting PC and distinguishing benign from tumor lesions in patients over 50 yrs with a serum PSA level between 4 and 10 ng/mL and normal DRE.

Several clinical studies have shown that the PHI test is a stronger predictor of a positive biopsy compared to serum PSA or its individual components for the detection of any PC and csPC, particularly in patients with total PSA levels between 4 to 10 ng/mL [8]. In a meta-analysis of 28 studies including 16.762 patients, the PHI test showed a pooled sensitivity and specificity of 89% and 34%, respectively, for the detection of PC and a pooled sensitivity and specificity of 93% and 34% for the detection of HGPC [9]. 

It is of clinical importance to note that, depending on the cutoff value used, the PHI test improves the diagnostic accuracy for detection of PC and csPC over serum PSA and subsequently helps to reduce unnecessary biopsies (by 15% to 45%) or detection of insignificant PC, it also misses a substantial number of PC (up to 10% at a cutoff of 25) [10]. 

Two studies addressed the potential clinical utility of the PHI test in helping urologists in their decision to perform prostate biopsy in patients with serum PSA between 4 and 10 ng/mL and normal DRE. In the single-center prospective study conducted by Tosoian et al., the use of the PHI test in the clinical assessment was associated with a 9% decrease in prostate biopsies, while the rate of HGPC was unchanged [11]. No patient with a PHI score < 21.2 had csPC, whereas 76% of patients with a PHI score ≥ 55 were diagnosed with GS ≥ 7. A cutoff value of 27 discriminated between csPC and insignificant PC for patients with prior mpMRI and PIRADS scores ≤ 3. The authors also showed that PHI score increased with higher pathological GS (*p* = 0.002) and pathological stage (*p* = 0.001), and found that PHI density calculated using the formula PHI score/prostate volume is a promising biomarker for the detection of csPC (a cutoff value of 0.43 had a sensitivity of 0.98 and a specificity of 0.38). In the other recently published multicenter prospective study conducted by White et al., the PHI test was significantly associated with a 23.9% reduction in prostate biopsies and influenced the urologist’s biopsy decision management plan in 72.5% of cases. According to the responses to the proposed questionnaire used to assess whether the PHI test impacted the biopsy decision compared with the previous management plan of the same urologist based on routine TRUS-guided biopsy, urologists were more likely to recommend a biopsy based on an intermediate to high-risk PHI score (phi ≥ 36) [12].

Additionally, the predictive role of the PHI test for a positive biopsy was shown in combination with ERSPC RC, MRI, or other biomarkers. Thus, PHI-based ERSPC calculators outperformed the PSA-based RC for predicting PC and csPC in several studies. The studies of Gnanapragasam et al. demonstrated that the PHI test was not only a stronger predictor of positive MRI, but the combination of PHI with MRI improved the prediction of csPC (AUC = 0.84) [13,14]. At the cutoff value of 30, the PHI test reduced the rate of biopsies performed in patients with suspicious mpMRI lesions by 25% (while missing 6% of csPC), and the rate of biopsies performed after using the combined results from both the PHI test and MRI by 40%. Furthermore, it was shown that the combination of PHI test and MRI in the re-biopsy setting missed only 1 of 21 csPC (at a PHI test cutoff value of 35, it had a sensitivity of 0.99 (0.94–1), specificity of 0.17 (0.12–0.23), a PPV of 0.38 (0.32–0.44), and negative predictive value (NPV) of 0.97 (0.84–1) [13]. Therefore, these studies provide scientific evidence that the PHI test and MRI are complementary before performing a prostate biopsy. In a prospective study performed on 177 patients with PC treated by RP, Francesco Gentile et al. showed that an artificial neural network-based approach combining mpMRI and PHI could predict csPC at initial diagnosis with a sensitivity of 80% and a specificity of 68%. This method could be helpful in the individualized management of these patients [15]. In another study, the combination of PHI and Proclarix (a risk score based on thrombospondin-1, cathepsin D, total PSA, free PSA, and patient age) had a significantly higher diagnosis accuracy for csPC compared to the individual tests alone ((AUC for biopsy grade endpoint: 0.82 (95% CI: 0.77−0.87), AUC for pathological grade endpoint: 0.84 (95% CI: 0.80−0.88)) [16].

Some studies documented the role of the PHI test as a predictor of unfavorable pathological findings in radical prostatectomy (RP) specimens. However, whereas the PHI test predicted a pT3 stage or a pathological GS ≥ 7, it did not improve clinical decision-making [17].

The use of PHI test is limited in clinical practice by the lack of an appropriate cutoff value, based on strong scientific evidence, for biopsy decision-making, its use in particular settings (prebiopsy/re-biopsy settings, with or without mpMRI, before or after mpMRI), and its cost-effectiveness [18]. Although the PHI test is mentioned in current guidelines to improve the strategy of PC management, its role remains to be fully determined in conjunction with patient cancer history, RC, and MRI.

#### 2.1.2. 4Kscore (OPKO Lab, Nashville, TN, USA)

The 4Kscore combines serum levels from a panel of four kallikrein prostate-specific biomarkers (total PSA, PSA free, intact PSA, and human kallikrein protein 2 (hK2)) with clinical parameters (age, prior biopsy status, and DRE) in an algorithm (OPKO algorithm) to predict aggressive forms of PC (GS ≥ 7) and discriminate between indolent (low-risk 4Kscore between 1–7.5) vs. csPC (high-risk 4Kscore of ≥20) in patients with suspicious PSA level or DRE at the initial or repeat set of biopsy [19]. In fact, 4kscore gives an individualized risk prediction score (a probability of detecting aggressive PC from 1 to 100) that helps urologists in biopsy clinical decision-making. For example, urologists can consider biopsying a younger man with or without additional PC risk factors at a cutoff value of <7.5 and an older man with significant comorbidities at higher cutoff values. 

The clinical usefulness of the 4Kscore in the initial diagnosis process of PC, particularly in the decision to perform a prostate biopsy or re-biopsy in patients (unscreened or prior screened) thought to harbor aggressive PC (GS ≥ 7), and its additional impact in avoiding unnecessary biopsies have been demonstrated in numerous trials and clinical utility studies [20,21,22,23]. A systematic review and meta-analysis conducted by Zappala et al. in 2017 to evaluate its predictive accuracy for detecting aggressive PC showed a pooled discrimination (AUC) value of >0.8 across multiple US and European studies when compared with standard-of-care models (based on age, PSA, and DRE) and with respect to changes in the algorithm and contemporary guidelines (those reflecting changes in biopsy procedure or Gleason grading report system) [24]. In the first prospective US study, which enrolled 1.012 patients in the validation phase, the authors reported a predictive accuracy of 0.82 and showed, for clinical utility purposes, that using a 4kscore cutoff value of 7.5 for predicting GS ≥ 7, 36% of prostate biopsies could be avoided while only 1.7% of diagnosis of csPC would be delayed (majority GS 3 + 4 and no GS ≥ 8) (overall a 30% to 58% reduction in unnecessary biopsies while only 1.3% to 4.7% of GS ≥ 7 were missed). The decision curve analysis performed to investigate the net clinical benefit (defined by benefit due to true positive results versus harm due to false negative results) showed a higher AUC compared with the common clinically used PCPTRC v 2.0 across all risk cutoffs. In another prospective clinical trial conducted in 6.129 patients with PSA ≥ 3 ng/mL undergoing 10 core biopsies, the 4kscore showed higher predictive accuracy for any PC or HGPC compared with a model based on PSA or age. Moreover, the authors showed that a cutoff of 6 for predicting HGPC would avoid 428 unnecessary biopsies (from 1000 screened patients), detect 119 HGPC (from 1000 patients), and delay diagnosis in 14 patients (from 133) [21]. A meta-analysis performed by Voigt et al. in 2014 using data from more than 8.500 patients showed a statistically significant improvement of 10% to 13% in the urologist’s ability to correctly identify any PC and of 8% to 10% to correctly identify any HGPC (based on the discrimination accuracy of the 4kscore compared with any of its individual parameters). In addition, this meta-analysis showed that 48% to 56% of prostate biopsies performed for PSA ≥ 3 ng/mL could be avoided and the rate of delayed diagnosis of HGPC or any PC was 0.63–0.70% and approximatively 7% respectively [25]. In another clinical utility study, Konety et al. showed that performing a 4kscore test in urological practice before a patient’s referral to a biopsy procedure (prebiopsy settings) and stratifying patients into low (<7.5%), intermediate, and high risk (≥20%) groups influenced the biopsy decision in 88.7% of cases and led to a 64.6% reduction in prostate biopsies [22]. Also, in this study, the 4kscore risk groups were associated with biopsy findings on pathological specimens. 

A meta-analysis conducted to compare the accuracy in the diagnosis of PC and clinical usefulness of 4kscore and PHI tests revealed that both tests performed similarly in detecting overall PC and HGPC (GS ≥ 7) (AUC of 0.82 and 0.81 for PHI and 4kscore, respectively; for HGPC, the pooled sensitivity was 0.93 (for PHI) and 0.87 (for 4kscore) while the pooled specificity was 0.34 (for PHI) and 0.61 (for 4kscore) [9]. 

The clinical utility of 4kscore has also been demonstrated in treatment decision-making such as in predicting the reclassification of PC patients on AS or the unfavorable pathological findings on RP specimens. In a cohort of 392 screened patients who were diagnosed with PC at PSA ≥ 3 ng/mL and underwent RP, 4kscore showed a higher predictive accuracy for detecting aggressive pathology (defined by pT3-T4, extracapsular extension, tumor volume > 0.5 cmc, or Gleason grade ≥ 4) compared to a clinical model (based on age, stage, PSA, and biopsy Gleason) (AUC 0.84 versus 0.81). In addition, the authors reported that using the 4kscore would avoid 14% of unnecessary surgeries [26].

Other authors showed the usefulness of the 4kscore in predicting more significant clinical outcomes, such as the risk of progression to metastasis or death from PC. In a population-based case-control study that included 40.379 patients, Stattin et al. showed that 4kscore improves the prediction of distant PC-metastases occurring 10 and 20 years later. The study revealed that in 50 and 60-year-old patients with a PSA > 2 ng/mL, AUC for distant metastasis was improved with the 4kscore compared with PSA alone (0.75 for total PSA alone vs. 0.86 for 4kscore at age 50 and 0.805 vs. 0.875 at age 60). Moreover, a low-risk 4Kscore predicted a low risk of developing PC-metastasis. For example, a 60-year-old patient with a PSA ≥ 3 ng/mL and a 4Kscore < 7.5% has a risk of 0.2% and 1.8% of developing distant metastasis by 10 or 15 years later [27]. Their data support a risk-based adapted strategy for PC screening/early detection based on using 4kscore similar to recommendations proposed recently by EAU experts. 

Furthermore, to improve the biopsy decision-making protocol and facilitate the detection of csPC, the 4kscore was investigated in combination with RC or MRI. A recent prospective study that enrolled 2.872 men who underwent an initial prostate biopsy for a PSA level ≥ 3.0 ng/mL showed a significant improvement in the predictive performance of csPC for the combination of 4Kscore with the Rotterdam Prostate Cancer Risk Calculator (RPCRC, including age, PSA value, DRE abnormalities, and DRE based prostate volume estimates) (AUC = 0.89; sensitivity of 88% and specificity of 71% at a 5% risk cutoff) compared to 4Kscore (AUC = 0.88; *p* < 0.01) or RPCRC (AUC = 0.87; *p* < 0.01). In addition, depending on the cutoff used, the combined 4Kscore-RPCRC model reduced the number of unnecessary prostate biopsies by 60–80%, and missed only 1–2% of csPC with an additional net reduction of 3.3–7.2 biopsies per 100 patients. However, the clinical benefit must be weighed against additional costs associated with this model [28]. 

The clinical utility of 4kscore in combination with MRI was reported in a recently published study of 574 patients who underwent systematic or combined MRI/TRUS fusion biopsy for 4kscore test > 7%, abnormal DRE or PI-RADS score ≥ 3 according to PIRADS v.2. The authors developed and validated a nomogram incorporating age, prostate volume, prior negative biopsy, DRE findings, 4kscore and PI-RADS score that showed an AUC of 0.88 for predicting csPC. When a prediction model with a threshold of 30 was used, the authors found that 30% of overall biopsies, 41% of benign biopsies, and 19% of indolent PC could be avoided and 9% of csPC could be missed [29].

Other studies reported the highest benefit of using a combined strategy of 4Kscore at a 10% threshold with selective MRI [30].

In conclusion, the 4Kscore could be a significant predictor of csPC within contemporary biopsy-decision protocols. Moreover, the 4kscore test has been shown to have a positive socio-economic impact by reducing healthcare costs system and improving patients’ quality of life [31].

Although it is not yet FDA approved (but CLIA lab) and no appropriate cutoff value has been validated for decision to perform a prostate biopsy, the 4kscore has been included in the NCCN Prostate Cancer Early Detection guidelines and the EAU guidelines since 2015 to be considered when the patients and/or their urologists wish to further assess the risk of harboring HGPC (Gleason score ≥ 3 + 4, Grade Group 2 or higher). Most of the data presented on 4K testing can be challenged by the mpMRI sensitivity and NPV value, therefore studies comparing 4Kscore with MRI or a combination of them are needed.

### 2.2. Urinary Biomarkers

#### 2.2.1. Prostate Cancer Antigen 3 (PCA3) Assay (Progensa^TM^ PCA3 Assay; Gen-Probe, San Diego, CA, USA)

This is a quantitative RT-PCR-based test that measures concentrations of mRNA PCA3 and mRNA PSA in the first void urine sample following DRE to provide a score (0 to >100) using their ratio (urinary mRNA PCA3/mRNA PSA × 1000). PCA3 is a gene that expresses a noncoding prostate tissue-specific RNA that is overexpressed in >95% of PCs (not in normal prostate tissue) independently of prostate volume and serum PSA value. mRNA PCA3 plays an essential role in prostate cancer cell survival by modulating androgen receptor signaling. Numerous studies have shown that PCA3 score improves diagnosis of PC (including of csPC), being most useful in determining which patients should undergo or avoid a repeat biopsy. In 2012, the FDA approved the PCA3 test to be used in the algorithm of re-biopsy decision-making among patients with suspicious PSA values and normal DRE. However, the PCA3 score showed a better predictive accuracy of PC diagnosis compared with PSA, free PSA, or PSA density at initial or repeated sets of biopsies with an acceptable sensitivity and specificity [32,33,34]. To date, several articles tried to determine the optimal PCA3 cutoff value for predicting a positive biopsy result but no globally accepted cutoff value could be established. Some studies reported a sensitivity of 64% and a specificity of 76% for a cutoff value of 35, while in other articles a PCA3 score cutoff value of 25 showed a sensitivity of 78%, specificity of 57%, NPV of 90%, and PPV of 34% [35]. Lower scores were associated with a lower likelihood of detecting PC at biopsy and therefore ruled out csPC. Additionally, increasing PCA3 scores were associated with a high probability of detecting any PC and HGPC. 

Hansen et al. developed and internally validated a PCA3-based nomogram to predict the risk of PC and csPC at initial prostate biopsy [36]. Their model (at PCA3 cutoff 21) had a higher predictive accuracy for detecting any PC (80.7% vs. 73.6%) and a higher net clinical benefit compared with a base clinical model (for example using a nomogram thresholds value of 30 would avoid up to 55% of unnecessary prostate biopsies while missing only 2% of csPC). 

In 2014, Wei et al. evaluated in a prospective validation trial the benefit of different PCA3 scores (<20, 20–60, >60) for PC detection among patients scheduled for initial or repeat biopsy based on elevated or increasing PSA, %fPSA < 15%, family history, prior ASAP or HGPIN or abnormal DRE [37]. For the initial biopsy setting, PPV (positive predictive value) was 80% (95% CI, 72–86%) at a cutoff value > 60 (sensitivity 0.42 and specificity 0.91). The use of PCA3 score at a cutoff value < 20 in the repeat biopsy setting (in patients with prior negative biopsy results) was associated with an NPV of 88% (sensitivity 0.76 and specificity 0.52). Also, the authors showed that the PCA3 score improved the prediction accuracy of positive biopsy including the detection of csPC in addition to PCPTRC (in the initial setting AUC increased from 0.68 to 0.79 for any PC and from 0.74 to 0.78 for HGPC; in the rebiopsy setting AUC increased from 0.64 to 0.69 for any PC and from 0.74 to 0.79 for HGPC). Moreover, in this trial a PCA3 score < 20 would avoid almost half of prostate biopsies irrespective of PSA values (46% and 41% in the initial and rebiopsy settings respectively). However, 13% of csPC in those without prior biopsy and only 3% in the repeat biopsy would be missed. 

Ankerst et al. showed that the incorporation of PCA3 into the PCPTRC improved its discrimination accuracy for predicting HGPC (76.4% vs. 70%) and demonstrated only a marginal clinical net benefit at thresholds from 10% to 50% compared with PCPTRC alone [38].

The added value of the PCA3 score to another widely used RC, the ERSPC RC4, was evaluated in a study by Vedder et al. [39]. The authors showed that the predictive accuracy for detecting PC was higher for the combination model of PCA3 score > 10 with ERSPC RC4 than RC4 alone (AUC 0.73 vs. 0.70; *p* = 0.02). When the performance of PCA3 was compared with 4kscore among prescreened patients with PSA ≥ 3 ng/mL, AUC was higher for the 4kscore in a univariate model (0.63 vs. 0.56; *p* = 0.05) but similar for the combination of each test with ERSPC RC4 (0.80 for RC4 + DRE and 0.78 vs. 0.79 for RC4 + PCA3 and RC4 + 4kscore, respectively). Moreover, the decision curve analysis to evaluate the clinical usefulness of the combination models in reducing the number of biopsies showed an improved net benefit of PCA3 or PCA3 + 4kscore with RC compared with RC alone in all patients undergoing a biopsy if the threshold was > 9%. 

Other authors tried to compare PCA3 with PHI in predicting csPC, but the data is contradictory [9,40,41].

Furthermore, Tombal et al. showed that adding the PCA3 score at cutoff 20 to the best clinical judgment according to experts’ recommendations would reduce the number of rebiopsies, have an NPV of 99% for detecting csPC, and misses 8 cases out of 55 (compared with 7 using PCA3 at cutoff 20) [42].

Fenstermaker et al. evaluated the role of PCA3 in predicting PC among patients stratified by MRI-fusion-targeted prostate biopsy. The authors showed that PCA3 demonstrated a better predictive ability for PC detection among patients with a low suspicion score on MRI, and using the strategy of performing a biopsy in cases with a PCA3 score > 35 would miss only 4.9% of csPC (Gleason score ≥ 4 + 3) [43]. 

The PCA3 assay was included in EAU and NCCN guidelines for indication in rebiopsy of patients with at least one prior negative prostate biopsy and at high risk for PC detection.

#### 2.2.2. Michigan Prostate Score Assay (MiPS, Mlabs, Ann Arbor, MI, USA) 

This score was developed by and is commercially available through the University of Michigan Mlabs. This test incorporates serum PSA and two urinary biomarkers (PCA3 and TMPRSS2-ERG) in a logistic regression model that calculates a score for detecting PC and predicting HGPC (Gleason score ≥ 7) on prostate biopsy. TMPRSS2-ERG, the abnormal fusion between a transmembrane serine 2 protease gene (TMPRSS2) and E26 oncogene homolog (ERG) gena protease, has been found to occur with high frequency in PC since the early stages of disease; it has a high specificity of 93% and PPV of 94% for PC diagnosis in the prebiopsy setting [44]. 

Although several articles reported a significant predictive accuracy of MiPS for PC or HGPC detection compared with PSA (AUC 0.88 and 0.77 for PC and csPC respectively), only one study validated its performance in a cohort of 1.225 patients presenting for initial (80%) or repeat biopsy [45,46,47]. The authors showed that MiPS outperforms not only PSA but also PCPTRC in predicting PC or HGPC. Moreover, models that combine MiPS with PSA, PCA3, or RC (PCPTRC) showed significantly better AUC. Also, a net clinical benefit of the models incorporating MiPS on avoiding unnecessary biopsies, identifying, or delaying csPC (compared with standard criteria for biopsy) was shown across relevant risk cutoffs. 

While the MiPS assay and MiPS-based models demonstrated clinical benefit and superiority compared with standard strategies for biopsy-making decisions, the experts consider that this test needs further validation to be recommended in routine clinical practice. 

#### 2.2.3. Select MDX Assay (MDxHealth, Inc., Irvine, CA, USA)

This assay measures urinary mRNA concentrations of HOXC6 and DLX1 genes (associated with PC aggressiveness) from post-DRE urine using quantitative RT-PCR. 

The test showed significant predictive accuracy for csPC in patients undergoing initial biopsy (AUC 0.76, sensitivity 91%, specificity 36%, NPV 94%, PPV 27%) [48]. Moreover, van Neste et al. showed that combining Select MDX with serum PSA, PSA density, age, DRE, family history, and prior negative history of biopsy increased AUC to 0.9 (or 0.86 in the validation cohort) in predicting the risk of csPC among patients with PSA > 3 ng/mL. Also, the authors validated two risk models (with or without DRE) for clinical utility in an independent cohort, and using decision curve analysis showed net clinical benefit in identifying csPC relative to PCPTRC or PCPTRC + PCA3 while decreasing the number of unnecessary biopsies [49]. At a cutoff value ≥ 2.8 rs, 42% of biopsies could be avoided but misses 2% of csPC. Recently, Haese et al. validated the model in predicting csPC for biopsy-naive patients with PSA < 10 ng/mL and reported an AUC of 0.82 (sensitivity of 89%, specificity of 53%, NPV 95%) [50]. Likewise, Hendriks et al. demonstrated the diagnostic performance of Select MDX (44% detection rate of csPC, 38% biopsies, and MRI avoided, 10% of csPC missed) and evaluated its potential clinical use in different combinations with MRI for detection of csPC [51]. Although the MRI-only strategy showed the best net benefit, the strategy of testing all patients with Select MDX followed by performing MRI only for patients with a positive test (a risk score ≥ −2.8) resulted in 60% of biopsies being avoided (compared with 49% in the MRI-only strategy) while missing 24 cases of csPC (compared with 9 in the MRI-only strategy) and was considered by the authors the preferred strategy if MRI availability is limited. In other recent prospective multi-institutional studies, a first biopsy can be proposed for patients with PSA > 3 ng/mL and PIRADS score 4–5 or PIRADS score 1–3 followed by a positive Select MDX [52].

In expert opinion, the Select MDX test can be considered in biopsy-naive patients to avoid unnecessary biopsies and reduce the risk of detection of indolent PC and missing csPC.

#### 2.2.4. ExoDx Prostate Assay (INTELLISCORE) (EPI; Exosome Diagnostics, Cambridge, MA, USA)

This is a non-invasive RT-PCR test (not requiring DRE) that measures concentration levels of 3 urinary exosomal RNAs (ERG and PCA3 normalized to SPDEF) to calculate a risk score (range 0 to 100). This unique test (easy to collect, handle, and process) was shown to discriminate benign disease or low-risk (GS 6) (risk score < 15.6) from high-risk PC (GS > 7) (at risk score > 15.6), therefore helping clinicians in initial biopsy-decision-making among patients with a serum PSA level < 10 ng/mL. 

In a study conducted by McKiernan et al. in patients aged 50 yrs or older undergoing initial or repeat prostate biopsy due to suspicious serum PSA levels (range 2 to 20 ng/mL) or DRE, the discrimination accuracy was improved when standard of care clinical variables (PSA level, age, race, family history) were added to the ExoDx Prostate assay (from 0.74 to 0.77 in the training cohort and from 0.71 to 0.73 in the validation cohort) [53]. The clinical utility study performed in 2018 showed that at a cutoff level of 15.6, the test could avoid 26% of unnecessary biopsies while missing 7% of csPC (NPV 89%) [54]. More recently, McKiernan et al. demonstrated similar performance of the ExoDx Prostate assay in predicting HGPC in repeat biopsy settings and net benefit compared with PSA or ERSPCRC [55]. 

Based on the above-mentioned data and other clinical utility studies, the experts from EAU and NCCN consider that ExoDx Prostate assay can be an option for initial or repeat biopsy-decision-making [56].

### 2.3. Tissue Biomarkers

#### Confirm MDX Assay (MDxHealth, Inc., Irvine, CA, USA)

This assay is a PCR-based test (CLIA-certified, not FDA-approved) that uses prior negative prostate biopsy tissue to discriminate between true negative patients and patients harboring an undetected/occult PC. This assay is based on epigenetic changes associated with the risk of PC development existing/occurring adjacent to tumor foci (halo effect) and evaluates quantitatively the DNA methylation level of GSTP1, APC, and RASSF1. 

In two validation studies, the NPV was 88% and 90%, meaning that the Confirm MDX assay is a significant predictor for the detection of PC on repeat biopsy [57,58]. Data regarding its clinical utility and the test’s performance in predicting csPC are expected. 

While the EAU made no recommendation regarding the routine clinical use of the Confirm MDX assay, the experts from NCCN Guidelines Version 2.2021 Prostate Cancer Early Detection believe that the Confirm MDX assay can be an option for patients considering repeat biopsy to reduce the rate of unnecessary repeat prostate biopsies.

### 2.4. Implications of Increased Genetic Risk

Family history of PC and family or personal history of other hereditary cancers or known high-risk gene mutations for PC are among the most important risk factors for PC development, estimated in the range of 5–15%.

Nowadays, uro-oncological guidelines consider patients with known or suspected germline mutations in PC predisposition genes as a group at particularly high risk of developing aggressive forms of PC who may benefit from early PC detection and specific therapy. However, the best screening or biopsy-based strategies are unknown. Experts recommend germline testing in patients with a family history of hereditary PC criteria or other cancer syndromes (genes to be considered—BRCA2, BRCA1, ATM, CHEK2, PALB2, HOXB13, MLH1, MSH2, MSH6, PMS2, NBN, BRIP1, and TP53) and annual screening for BRCA2 carriers or other germline mutations starting at age 40 yrs and at the age corresponding to 10 yrs prior to the age of youngest family diagnosis of PC. Biopsy-decision is based on age-adjusted median serum PSA level (the higher the median, the greater the risk), DRE, or imaging.

## 3. Biomarkers for the Management of Clinically Localized Prostate Cancer

Treatment options for patients diagnosed with clinically localized PC include AS, surgery (RP), or radiation therapy (RT). However, in the management of these patients, clinicians should incorporate risk factors for disease recurrence or progression to metastasis. Although there are numerous pretreatment risk stratification tools available, there is an ongoing debate as to which of these is better for predicting the risk of progression. Moreover, current uro-oncological guidelines suggest that germline testing (e.g., for mutations in BRCA1, BRCA2, ATM, and CHEK2) or tissue-based molecular assays (Decipher, Oncotype, Prolaris, or ProMark) (Table 3) may also be considered in the optimal selection of an initial treatment option for curative intent or to predict adjuvant therapy, especially when they will influence decision-making. Nevertheless, the expert recommendations differ. NCCN guidelines state that patients with low risk or favorable intermediate-risk and a life expectancy > 10 yrs may consider the use of Decipher, Oncotype, or Prolaris while patients with unfavorable intermediate-risk or high-risk and with life expectancy > 10 yrs may use Decipher or Prolaris. In addition, Decipher may be used to inform adjuvant treatment after RP if adverse features are reported in the pathology. EAU guidelines state that these four biomarkers can be considered to improve the accuracy for identifying patients with AS (or patients with csPC) who may benefit from curative treatments. However, more validation studies are needed to consider these biomarkers in routine clinical practice.

### 3.1. Decipher (GenomeDx, San Diego, CA, USA)

This was developed and validated as a 22 RNA biomarker genomic classifier (GC) to predict early clinical metastasis and PC-specific mortality after biochemical recurrence in patients diagnosed with localized PC and treated with RP. It measures tissue expression of 22 coding and non-coding RNA gene sequences using microarray technology to calculate a score ranging from 0 to 1 [59]. 

The prognostic role of Decipher in post-RP settings was studied in most of the data published. First, the Decipher test was validated as a significant and better predictor of metastasis (compared with clinicopathological-based models) in high-risk post-RP patients (AUC 0.79 for predicting metastasis occurrence at 5 yrs) and time to metastasis. In addition, combination with conventional nomograms (Stephenson or CAPRA-S nomograms) increased its predictive accuracy [60,61,62,63]. A higher score (>0.6) was associated with a higher risk for metastasis. On the contrary, a low score was associated with prolonged cancer control, irrespective of the timing of RT initiation. 

Second, the Decipher test was shown to predict PC-specific mortality (AUC 0.78). In one validation study, a Decipher score > 0.6 was associated with an increased risk of PC-specific mortality within 10 yrs in patients with adverse pathological features after RP (pT3, pN1, positive margins, or Gleason score > 7) [64]. 

In addition, the Decipher test demonstrated a predictive role in improving decision-making post-RP regarding the indication of RT. Dalela et al. evaluated a risk model combining Decipher with available clinicopathological variables in patients with adverse features on RP specimens [65]. The authors showed that patients with more than 2 poor prognostic variables and higher Decipher scores had greater benefit (low clinical recurrence) with upfront adjuvant RT (versus initial observation) compared with those with < 2 risk variables and a low Decipher score, who could safely avoid unnecessary RT. Further, the PRO-IMPACT study evaluated the impact of the Decipher test on clinical decision-making for RT post-RP (adjuvant RT versus salvage RT). The initial treatment decision was changed in 18% and 32% of patients in the adjuvant RT and salvage RT groups, respectively, after applying the Decipher test [66]. In addition, Den et al. showed that patients with a low Decipher score (<0.4) had similar incidences of metastasis post-RT, regardless of the timing of post-RP RT, while patients with high Decipher scores ≥ 0.4 are best treated with adjuvant RT (5 yrs cumulative incidence of clinical metastasis was 6% for adjuvant RT compared with 23% for salvage RT) [67]. Also, the clinical utility of assessing the impact of Decipher in the post-RP setting is important. Marascio et al. demonstrated the clinical benefit and clinical utility of the Decipher test in a prospective study [68]. The authors showed that patients with high-risk scores who were treated with recommended adjuvant RT had only a 3% of a 2-year biochemical recurrence rate (compared with 25% of patients who did not follow the recommendation), and Decipher testing changed the recommended treatment in 39% of patients, with 3 tests needed to be performed to change one treatment decision. Recent data from the RADICALS-RT phase III clinical trial showed that routine administration of adjuvant RT post-RP is not appropriate due to the high toxicity, and this treatment method should be used as salvage radiotherapy only. Moreover, the unpublished final data of the aforementioned trial confirms the initial results. Thus, the role of the Decipher test in the decision-making of adjuvant RT post-RP should be revised due to the data from the RADICALS-RT phase III trial.

In 2017, Nguyen et al. published the first report on Decipher’s ability to predict distant metastasis and showed that patients with a Decipher high-risk score (>0.6) had a high rate of cumulative incidence of metastasis (20%) compared to those with a score ≤ 0.2 [69]. Another study demonstrated the predictive role of Decipher for ADT-decision-making when given in combination with RT post-RP and suggested that hormonal therapy (bicalutamide) can be omitted in patients receiving salvage RT with lower Decipher scores [70].

It is of clinical importance that the Decipher test performed on biopsies cores was able to predict adverse pathology at RP (NPV 96% for cutoff 0.2), thus being a useful risk stratification tool (predictor) in patients considering AS [71]. 

In conclusion, the Decipher test demonstrated a valuable prognostic and predictive value in biopsy or post-biopsy settings (particularly post-RP).

### 3.2. Oncotype Dx (Oncotype Dx Genomic Prostate Score, Genomic Health, Redwood, CA, USA)

This is a quantitative RT-PCR assay specially conceived to be performed on small samples from prostate needle biopsies. It measures the expression of 12 PC genes linked to 4 prostate oncocarcinogenesis pathways and 5 reference genes that are combined to calculate the Genomic Prostate Score (GPS) (range 0–100). This assay was studied using prostate needle biopsies in patients with low- to intermediate-risk proposed for AS to predict adverse pathological features on RP specimens (grade group ≥ 3 or pT3) or aggressive behavior of clinically localized PC (metastasis, PC death) [72,73]. 

In the validation study of Klein et al. [71], GPS was a significant predictor of higher grade and higher disease stage at RP (OR per each 20 GPS units: 2.3 (95% CI, 1.5–3.7; *p* < 0.001) for high-grade and OR per each 20 GPS units: 1.9 (95% CI, 1.3–3.0; *p* = 0.003) for high stage). While similar findings were reported by other authors, recent data did not conclude that GPS improved management decisions in low or intermediate risk groups of patients considering AS [74,75,76,77]. Moreover, one recent randomized trial addressing the impact of GPS on clinical decision-making showed that integrating GPS in urological practice would negatively affect patient’s decision to choose AS [78]. 

Limited data on GPS use as a prognostic tool for biochemical recurrence or progression after RP (metastasis, PC death) are available. In 2015, Cullen et al. showed that GPS predicted biochemical recurrence-free survival rate and time to metastasis [75]. Another retrospective study consisting of 279 patients with low- to high-risk disease treated with RP between 1995 and 2010 showed that higher GPS was strongly associated with shorter time to metastasis and time to PC-death and improved AUC for predicting long-term outcome (at 10 yrs) when combined with CAPRA (from 0.78 to 0.84; *p* < 0.001) [79].

In expert opinion, GPS, in conjunction with routine clinical assessment parameters, can be considered to stratify and counsel patients who are most likely to benefit from AS management.

### 3.3. Prolaris (Myriad Genetic Laboratories, Salt Lake City, UT, USA)

This is a quantitative RT-PCR assay that measures expression levels of 31 cell cycle progression genes normalized to 15 reference genes to provide a cell-cycle progression score (CCPS, range 0–10) determined as described by Cuzick et al. in 2011 [80]. This assay (available in the USA and Europe) can be performed on small prostate tissue from biopsy, transurethral resected, or RP specimens. Prolaris was developed to predict the aggressiveness of localized PC by providing a 10 year-risk assessment of biochemical recurrence and metastasis after RP or RT or 10 year-PCSM for patients considering AS. It can be used in localized PC as a risk stratification or prognostication tool post-biopsy or post-RP to help in the further therapeutic decision-making process.

The prognostic utility of this test was first reported in 2011 by Cuzick et al. in a retrospective analysis of 2 cohorts of patients undergoing RP (prostatectomy cohort) and watchful waiting after transurethral resection of the prostate (post-biopsy cohort) [80]. In multivariable analysis, CCPS was the strongest predictor of biochemical recurrence in the prostatectomy cohort (HR for a 1-unit change [doubling] in CCP 1.77, 95% CI 1.40–2.22; *p* = 4·3 × 10^−6^) and of PCSM in the post-biopsy cohort (HR for a 1-unit change [doubling] in CCP 2.57, 95% CI 1.93–3.43; *p* = 8·2 × 10^−11^). Their results were validated in several retrospective papers.

In the post-RP setting, Cooperberg et al. showed that CCPS was associated with biochemical recurrence independent of the CAPRA-S risk assessment score (Cancer of the Prostate Risk Assessment post-Surgical) (HR for a 1-unit increase in CCP score of 1.7, 95% CI, 1.3 to 2.4, with adjustment for CAPRA-S) [81]. Moreover, the combined CCPS and CAPRA-S scores performed better than each individual score, and the authors concluded that the Prolaris test can be used as a risk stratification tool for patients with localized PC, including the low-risk groups defined by a CAPRA score ≤ 2. Another more recent post-RP validation study showed a strong correlation between CCPS and a combined CCPS and CAPRA score with 10-year risk of progression to metastatic disease (HR for a 1-unit change in CCP score 2.21, 95% CI 1.64–2.98, *p* = 1.9 × 10^−6^; HR for a 1-unit change in combined score 3.63, 95% CI 2.60–5.05, *p* = 2.1 × 10^−16^) [82].

In the post-biopsy setting, Bishoff et al. showed at multivariate analysis that biopsy CCPS was a significant predictor of biochemical recurrence (HR for a 1-unit change in CCP score 1.47, 95% CI 1.23–1.76, *p* = 4.7 × 10^−5^) and metastatic disease (HR for a 1-unit change in CCP score unit 4.19, 95% CI 2.08–8.45, *p* = 8.2 × 10^−6^) after RP in a multicenter retrospective study [83]. In another validation report by Cuzick et al. CCPS and combined CCPS and CAPRA scores were predictive for PCSM (HR 1.77 and 2.17, respectively, *p* < 0.05) in a cohort of patients with clinically localized PC undergoing conservative management [84]. The clinical utility of the biopsy CCP score was also evaluated for predicting biochemical recurrence in localized PC managed by external beam RT. In the study of Freedland et al., CCPS was a significant predictor of biochemical recurrence (HR for a 1-unit change in CCP score 2.11, 95% CI 1.05–4.25, *p* = 0.034 in multivariate analysis) and was associated with 10 years-PCSM (HR for a1-unit change in CCP score 3.77, 95% CI 1.37–10.4, *p* = 0.013) [85]. 

Sommariva et al. reported in their metanalysis a pooled HR for a 1-unit change in CCP score of 2.08 for PCSM and 1.634 for biochemical recurrence [86]. Moreover, the authors reviewed the studies investigating the impact of Prolaris on the clinical decision-making process and showed that CCP testing can lead to the reclassification of patients into risk groups and a change in treatment recommendation (reduction or increase of RP or RT procedures, particularly among low-risk groups of patients).

Current uro-oncological guidelines state that the Prolaris test can be used as a decision tool for the initial management of localized PC, particularly when the test report may or will affect treatment decisions. Such a situation can include a better classification of a patient into a risk group with an immediate impact on the oncological outcome as well as the patient’s quality of life.

### 3.4. ProMark Test (Metamark, Cambridge, MA, USA)

This test is a quantitative immunofluorescent assay performed on biopsy specimens that measure the expression levels of 8 proteins to provide a score (range 0 to 1) reflecting the aggressiveness of PC (the higher risk score correlates with a higher risk of aggressiveness). The test was designed to predict the probability of HGPC (Gleason > or equal to 4 + 3) and non-organ-confined PC on RP (unfavorable pathology) or PCSM and is indicated in patients diagnosed with low-risk localized PC considering AS. After studying numerous proteins and their functional status (activity state), Shipitsin et al. selected 12 protein biomarkers that were able to predict PCSM [87]. Blume-Jensen et al. refined the assay to 8 biomarkers and demonstrated in 381 cases that the assay was able to predict HGPC in patients undergoing RP [88]. At risk scores > 0.8, the predictive value for unfavorable pathology (pathological Gleason ≥ 4 + 3 or non-organ-confined (T3a, T3b, N, or M)) was 76.9% across all risk groups. Increased assay scores were correlated with a decreased incidence of favorable outcomes in all patients. A second validation study was conducted by the same authors on 276 cases, proving the ability of the assay to differentiate favorable from non-favorable outcomes (AUC, 0.68; *p* < 0.0001; OR, 20.9) and GS-6 pathology (defined by pathological Gleason = 3 + 3 and localized ≤ T3a) versus non–GS-6 pathology (defined by surgical Gleason ≥ 3 + 4 or nonlocalized (T3b, N, or M)) (AUC, 0.65; *p* < 0.0001; OR, 12.95). 

To conclude and reflect the current guidelines, ProMark test is recommended as an independent risk assessment that can improve the clinical decision-making process in patients diagnosed with low-risk localized PC and at least 10 years life expectancy.

## 4. Biomarkers for Advanced Prostate Cancer

Androgen Deprivation Therapy (ADT) is usually a highly effective treatment for patients with advanced PC for a substantial period of time. Although ADT decreases serum PSA levels and even produces objective tumor responses, virtually all patients will progress and develop metastatic disease. Contemporary research studies showed that PC progression from an androgen-dependent (hormone-sensitive) to an androgen-independent (castration-resistant) state is related to abnormalities that affect androgen receptor (AR) signaling, DNA repair pathways, and other molecules that are involved in the regulation of cell survival and apoptosis (Table 4). Therefore, researcher efforts were directed to identify abnormalities involved in PC progression and metastatic behavior, both for the purpose of obtaining prognostic factors for aggressive behavior and potential targeted therapy. 

Since the prevalence of germline or somatic mutations in patients with aggressive PC is high and may affect therapeutical decisions, consensus-based guidelines from the NCCN, American Urological Association, and EAU recommend genetic counseling and germline testing for a panel of genes including mutL homolog 1 (MLH1), mutS homolog 2 (MSH2), mutS homolog 6 (MSH6), and postmeiotic segregation increased 2 (PMS2; for Lynch syndrome); breast cancer susceptibility gene 1 and 2 (BRCA1, BRCA2); ataxia telangiectasia mutated (ATM); partner and localizer of BRCA2 (PALB2); and checkpoint kinase 2 (CHEK2) for patients diagnosed with high-risk, very high-risk, regional, or metastatic PC. Although the choice of treatment depends on several factors such as the site and extent of disease involvement, the presence or absence of symptoms, or prior systemic treatments, several genetic biomarkers were studied and validated for their prognosis utility or predictive ability to identify subsets of patients with significant responses to specific drugs, e.g., a poly[ADP-ribose] polymerase inhibitor (PARP inhibitor) for those with homologous recombination repair (HRR) deficiency or pembrolizumab for tumors associated with deficient mismatch repair [dMMR]/high levels of microsatellite instability [MSI-H].

### 4.1. HRR Deficiency

HRR deficiency is a predictive biomarker for response to PARP inhibitors and platinum-based agents. One treatment strategy for metastatic castration-resistant PC (mCRPC) recommended by NCCN v2.2021 relies on identifying tumors with defects in DNA damage repair (DDR) genes and DNA response mechanisms, such as in HRR, one major DDR mechanism initially described in other cancers (breast, ovarian, pancreatic). The identification of a germline or somatic mutation (mutation that arises within the tumor) in a DDR gene associated with HRR deficiency is of clinical interest for treatment implications in patients with metastatic PC, which is predictive of responses to PARP inhibitors and platinum-type drugs. The clinical utility of genetic testing in non-metastatic settings is currently unclear: data comes from retrospective studies, needs to be further validated, are mostly related to the prognostic role of the BRCA2 mutation, and suggests that BRCA2 carriers have an increased risk of biopsy upgrading, an increased risk of developing metastasis earlier post-RP or RT, and/or shorter time to castration resistance status [89].

The prevalence of germline HRR gene mutations was reported in up to 16.2% of patients with metastatic PC (compared with 3.8% and 8.9% in low-risk and high-risk localized PC), and the most frequently affected genes were BRCA2 (5.4%), CHEK2 (1.9%), ATM (1.6%), BRCA1 (0.9%), RAD51D (0.4%), PALB2 (0.4%), ATR (0.3%), PMS2 (0.3%), NBN (0.3%), and BRIP1 (0.2%) [90]. Somehow similar, the prevalence of somatic gene mutations was reported in 23% of mCRPC [91]. However, most of the studies exploring the prevalence of genomic alterations in DDR genes do not distinguish between the germline and somatic origin of the identified variant. 

Specimens that can be used for testing HRR gene mutations are blood or saliva samples (germline-only testing) and tumor tissue (from biopsy samples of a metastatic lesion or the biopsy samples from the primary tumor tissue). HRR gene mutations can be detected by different assays, including gene panels and next-generation sequencing (NGS) [92]. None of the NGS tests is validated or FDA-approved for germline assessment. Although the NGS assay for tumor tissue is the preferred approach, the limitations are related to the availability and quality of primary or metastatic tumor tissue. Also, the analysis of circulating tumor DNA is appealing, but the results are related to the high disease burden. It should be noted that germline and somatic testing are complementary (one test cannot substitute for the other; most somatic tests cannot distinguish between the germline or somatic variant; somatic testing alone may miss germline variants and vice versa) and therefore are both recommended. 

Olaparib (Lynparza, AstraZeneca)—The clinical benefit of olaparib in patients with mCRPC and HRR gene mutations vs. in those without identified gene abnormalities was reported by Mateo et al. in the TOPARP-A clinical trial (Trial of PARP Inhibition in Prostate Cancer), which enrolled 49 evaluable patients with progressive mCRPC after two prior therapeutical regimens (all patients had docetaxel), treated with olaparib (400 mg twice daily, oral use) until the occurrence of radiologic progression, unequivocal clinical progression, unacceptable side effects, withdrawal of consent, or death. DDR genes were assessed for all patients in tumor tissue samples obtained from metastatic site biopsies (bone marrow, nodes, and visceral metastasis in 43 patients) or archival tumor samples (in 6 patients) using the NGS assay [93]. Aberrations in DDR genes were detected in 33% of patients (16 patients), the most common of which were in BRCA2 and ATM. 88% of patients with a DDR gene had a response based upon one of the study criteria: an objective response according to RECIST version 1.1; a decrease in PSA level of 50% or the circulating tumor-cell count from 5 or more per 7.5 mL of blood measured at baseline using the CellSearch platform (Janssen Diagnostics). By contrast, only 3% of patients (1 patient) without an identified DDR gene had a response. Moreover, in the multivariable model for response, patients with an identified DDR gene had a higher response compared with those without any identified HRR abnormality (*p* < 0.001). Additionally, radiologic progression-free survival and overall survival were significantly longer in those with the biomarker-positive disease compared with biomarker-negative disease (9.8 versus 2.7 months and 13.8 versus 7.5 months, respectively).

In the TOPARB-B study, Mateo et al. prospectively validated the efficacy of olaparib in mCRPC with specific DDR abnormalities in tumor samples and consequently suggested the implementation of DDR testing by using targeted NGS in clinical practice to identify patients who are likely to respond to olaparib (as a predictive biomarker for responsiveness to olaparib) [94]. He underlined that the most clinical benefit was noted at a 400 mg dose (based on predefined criteria for success: at least 43% of patients in this dose cohort responded) and in the BRCA1/2 subgroup (based on the highest number of responses and the longest median radiographic progression-free survival). 

Phase 3 randomized PROfound trial (Study of Olaparib Versus Enzalutamide or Abiraterone Acetate in Men With Metastatic Castration-Resistant Prostate Cancer) evaluated olaparib (300 mg twice daily) in mCRPC patients with disease progression on abiraterone or enzalutamide (while previous taxane-based therapy was also permitted but not required) with at least one of 15 predefined HRR gene mutations detected in tumor samples by an investigational assay based on the FoundationOne CDx NGS test. 387 eligible patients were randomized in 2 cohorts: cohort A included 245 men with pathogenic variants in BRCA1/BRCA2, or ATM, and cohort B included 142 men with alterations in BRIP1, BARD1, CDK12, CHEK1, CHEK2, FANCL, PALB2, PPP2R2A, RAD51B, RAD51C, RAD51D, or RAD54L genes. The median radiological progression-free survival (the primary end point) in cohort A was significantly longer compared with the abiraterone/enzalutamide control group (7.4 versus 3.6 months, HR 0.34, 95% CI 0.25–0.47). Also, patients in cohort A had a higher objective response rate (33% versus 2%; odds ratio for an objective response, 20.86; 95% CI, 4.18 to 379.18; *p* < 0.001). Benefits persisted in the overall population (cohorts A and B) (median radiographic progression-free survival 5.8 versus 3.5 months, HR 0.49; 95% CI, 0.38 to 0.63; *p* < 0.001) [95]. In another phase 3 trial, overall survival was significantly improved for both cohorts with at least one alteration in BRCA1, BRCA2, or ATM (cohort A) or any of the other 12 prespecified genes (cohort B) (cohort A: median 19.1 versus 14.7 months; cohort B: median 14.1 versus 11.5 months) [96]. Analyses of efficacy in gene subgroup analysis revealed that the median overall survival for patients initially assigned to olaparib compared with those receiving abiraterone or enzalutamide was longer with BRCA2 mutations (24.8 versus 15.2 months; HR 0.59, 95% CI 0.37–0.95), while for BRCA1 mutations and those with ATM mutations, the corresponding values were 11.7 versus 9.4 months (HR 0.42, 95% CI 0.12–1.53) and 18 versus 15.6 months (HR 0.93, 95% CI 0.53–1.75).

Based upon the data from the PROfound trial, in May 2020 the FDA approved olaparib (300 mg twice daily) for oral use in patients with mCRPC progression following treatment with enzalutamide or abiraterone, irrespective of prior taxane-based therapy, and carrying a germline or somatic alteration in an HRR gene, determined on a positive test from tumor tissue (ATM, BRCA1, BRCA2, BARD1, BRIP1, CDK12, CHEK1, CHEK2, FANCL, PALB2, RAD51B, RAD51C, RAD51D, or RAD54), lymphocytes from blood (germline testing for BRCA1 or BRCA2), or plasma using a circulating tumor DNA assay (ATM, BRCA1, BRCA2).

Notably: (1) PPP2R2A was excluded from FDA drug approval due to the inferior activity of olaparib in this subset. Moreover, since the approval of olaparib includes patients harboring HRR genes that have not individually been shown to be predictive for sensitivity to olaparib, this can have negative consequences, such as that these patients with unclear clinical benefit will be exposed to drug toxicity and delays in receiving more effective treatments; (2) since prior taxane was not mandatory in the PROfound study, the NCCN v2.2021 guidelines recommend olaparib as a second-line treatment option after docetaxel or in subsequent lines of mCRPC therapy.

Rucaparib (Rubraca, Clovis Oncology Ireland Ltd., Building 2, Dublin Airport Central, Swords, Dublin)—The PARP inhibitor rucaparib received accelerated FDA approval in May 2020 only for BRCA1/2-mutated mCRPC based on data from the single-arm TRITON2 clinical trial. In the first analyses of this trial, 115 patients with mCRPC progression after one or two lines of an androgen receptor inhibitor (abiraterone or enzalutamide) or taxane-based therapy and harboring a germline or somatic BRCA1/2 mutation (as determined by germline testing, NGS of tumor tissue, or assay of circulating tumor DNA) received rucaparib 600 mg twice daily (orally) [97]. The objective response rate (primary endpoint) assessed by blinded independent review per modified RECIST v1.1 and Prostate Cancer Clinical Trials Working Group 3 (PCWG3) criteria was 43.5% (95% CI, 31.0% to 56.7%; 27 of 62). 88.7% of patients had at least stable disease, the majority by week 8. The PSA response rate was 54.8%, and the median radiographic progression-free survival was 9.0 months. The median time to PSA progression was 1.9 months (95% CI, 1.3 to 1.9 months). The investigators did not notice any difference in overall response rates for patients harboring a germline or a somatic alteration in BRCA2/BRCA1 and for patients with BRCA1 vs. BRCA2. A second analysis demonstrated similar clinical benefits in terms of objective and PSA response rate, irrespective of the assay used for testing the BRCA status (plasma, tissue, or genomic) [98].

Niraparib (Zejula, GlaxoSmithKline, Durham, NC 27701, USA) and Talazoparib (Talzenna, Pfizer Europe MA EEIG, Boulevard de la Plaine 17 1050 Bruxelles, Belgium) are two promising PARP inhibitors that are being evaluated in mCRPC. Niraparib demonstrated activity in patients with mCRPC and mutations in BRCA1/2 enrolled in one phase 1 and one phase 2 clinical trial [99,100]. Talazoparib showed higher clinical benefit in patients with mCRPC and BRCA1/2 mutations (objective response rates 46%/50%) compared with PALB2/ATM mutations (objective response rates 25%/12%) in the phase 2 TALAPRO-1 study [101]. 

In another multicenter study by Schmid et al., the efficacy of platinum-based chemotherapy was assessed among mCRPC patients with HRR mutations [102]. The authors showed that HRR mutations were associated with sensitivity to platinum-based chemotherapy (PSA50 response 47.1% vs. 36.1%, radiological soft tissue response 48.3% vs. 31.3%). Moreover, it was noted that 64% of PSA responses and 50% of radiological soft tissue responses were observed among the subgroups of patients with the BRCA2 mutation. However, these associations were not statistically significant. Median overall survival varies significantly among patients with HRR mutations: 15.2 months in cases with the BRCA2 mutation, 9.3 months for ATM, 4.1 months for BRCA1, and 4.9 months in cases with other DNA repair gene alterations.

The presence of mutations in DNA mismatch repair genes may have clinical implications not only for treatment with PARP inhibitors or taxane-based therapy but also for guiding decision-making regarding current treatment options for mCRPC. PROREPAIR-B, a prospective trial to evaluate the impact of ATM/BRCA1/BRCA2/PALB2 germline mutations on mCRPC clinical outcome, showed that the presence of a mutation in BRCA2 was an independent prognostic factor of cause-specific survival (CSS) (HR 2.11; *p* = 0.033) and suggested that BRCA2 can guide treatment selection (androgen signaling inhibitor vs. taxane-based therapy) or sequential therapy of mCRPC (CSS was better when patients were treated with first-line abiraterone or enzalutamide followed by taxane-based therapy) [103]. 

### 4.2. DNA Mismatch Repair and Tumor Mutational Burden

DNA mismatch repair is another DNA damage repair mechanism and mutation in one of the most relevant DNA mismatch repair genes (mutL homolog 1—MLH1, mutS homolog 2—MSH2, mutS homolog 6—MSH6, and postmeiotic segregation increased 2—PMS2) has been associated with responsiveness to anti-programmed cell death 1 (PD-1) immune checkpoint inhibitors. Tumors harboring MMR mutation genes are biologically characterized by a high level of microsatellite instability (MSI-H), and the vast majority have high levels of tumor mutational burden (TMB-H). However, not all cancers with TMB-H have dMMR and MSI-H. 

#### 4.2.1. Data for dMMR

The predictive value of DNA mismatch repair mutation genes or MSI-H on clinical efficacy of anti-programmed cell death 1 (PD-1) immune checkpoint inhibitors was demonstrated based on the results of five clinical trials (3 prospective and 2 retrospective) in which a total of 149 patients with 15 different types of metastatic or locally advanced, unresectable solid tumors and dMMR or MSI-H (colorectal—90 cases, endometrial—14 cases, other gastrointestinal cancers—35 cases, breast cancer 2 cases, prostate cancer 2 cases and bladder, sarcoma, thyroid, retroperitoneal, small cell lung cancer, renal cell cancer 1case each) received pembrolizumab after a median number of two prior lines of therapies. Mutations in DNA mismatch repair genes and MSI were assessed by immunohistochemistry (IHC) and PCR testing, respectively. The overall objective response rate (ORR) was 39.6%, with a complete response rate of 7.4% and a partial response rate of 32.2%. 78% of patients had a response duration of a minimum of 6 months. There were similar ORR between colorectal and non-colorectal patients [104]. Based on these results, in 2017, the FDA approved under its accelerated approval program and conditions the use of pembrolizumab for patients with ANY metastatic or unresectable solid tumors with MSI-H or dMMR, and disease progression after prior line/lines of therapy/therapies and no satisfactory available alternative treatment options. Although only 2 patients with mCRPC were included in the above-mentioned data analysis, of which one had a partial response and the other had stable disease for more than 9.8 months, and PC is not mentioned specifically in the FDA prescription, oncological experts support the use of pembrolizumab for mCRPC with dMMR or MSI-H mCRPC and disease progression after at least one line of systemic therapy. 

Additional data supporting the clinical efficacy of pembrolizumab in mCRPC are provided by two studies. In the phase Ib KEYNOTE-028 trial, which enrolled 23 patients with mCRPC and PD-L1 expression in ≥1% of tumor or stromal cells, the authors reported an ORR of 17.4% (including 4 partial responses and 8 stable diseases) and a durable response (median response duration was 13.5 months) [105]. The KEYNOTE-199 phase II trial investigated the efficacy of pembrolizumab in 3 cohorts of patients with docetaxel-refractory mCRPC [106]. The ORR per RECIST V1.1 criteria was modest (5% in the cohort comprising 133 patients with PD-L1 positive disease and 3% in the cohort comprising 67 patients with PD-1 negative disease) but durable (median duration of response was 16.8 months in the combined cohort analysis). Notably, the authors reported two complete responses (both in PD-L1 positive tumors) and 7 partial responses (5 in PD-L1 positive patients in 2 in PD-L1 negative patients). Moreover, the reported median overall survival of 9.5 months in cohort 1 and 7.9 months in cohort 2 is encouraging for this difficult-to-treat subset of patients. It is of clinical interest that in this study pembrolizumab demonstrated better clinical efficacity in a separate cohort of 59 patients with bone metastasis and no RECIST-measurable disease, irrespective of PD-L1 expression. However, exploratory biomarker analysis in the phase II KEYNOTE-199 trial did not demonstrate the association between the presence of mutations in DNA damage repair genes or dMMR genes and response to pembrolizumab, probably due to several limitations, including the number of samples, the number of responses, and the assay used for MSI testing [106]. 

#### 4.2.2. Data for TMB-H 

High levels of TMB, independent of MMR or MSI, are of clinical interest due to their potential predictive value for responsiveness to pembrolizumab as demonstrated in several retrospective studies. However, the most convincing data are provided by the phase 2 KEYNOTE-158 trial, which prospectively enrolled patients with different advanced incurable cancer types (no prostate cancer was enrolled) and investigated the association of high TMB with the efficacy of pembrolizumab [107]. TMB status was determined in tumor samples by using the NGS platform FoundationOne CDx assay. The cutoff value to define high levels of TMB and identify tumor sensitivity to pembrolizumab was prespecified at ≥10 mut/Mb (mutations per megabase). Thirteen percent of patients had high levels of TMB. ORR (the primary endpoint) was 29% in cases with high levels of TMB, compared with 6% reported for non-high levels of TMB. 

Largely based on the findings of this clinical trial, in June 2020, the FDA approved pembrolizumab for patients with any metastatic or unresectable solid tumor, including PC, that has a TMB ≥ 10 mut/Mb (as defined by the FDA-approved FoundationOne CDx diagnostic test used in KEYNOTE-158 trial), after progression following prior standard regimens, and who have no satisfactory alternative treatment options.

Notably, FDA prescriptions do not explicitly include PC. Moreover, there are other reports that failed to demonstrate the clinical usefulness of high levels of TMB in selecting patients for pembrolizumab, and the response varies across different tumor types. Additionally, it is unknown whether the cutoff TMB value of ≥10 mut/Mb is the appropriate threshold to define high TMB in PC. 

In our opinion, until validation studies provide convincing results for the sensitivity of high TMB PC patients to pembrolizumab, caution is advised in extrapolating the FDA prescription to PC. 

In May 2017, the FDA approved the anti-PD-1 immune checkpoint inhibitor pembrolizumab for the treatment of patients with solid tumors (including PC) and alterations in DNA damage repair pathway (dMMR and MSI-H), regardless of the tumor origin. In June 2020, the approval was extended to patients with TMB-H. Notably, the identification of dMMR or MSI-H in PC may indicate the presence of Lynch syndrome.

The reported prevalence of dMMR and MSI (assessed by NGS) in advanced PC is 1% and 1.7% respectively with a higher incidence in aggressive variants (Intraductal carcinoma and grade group 5 [108,109,110,111]. Regarding TMB, one prospective study reported a prevalence of 3% in patients with advanced PC. However, the majority (71%) of these patients had MSI-H [91]. 

For patients with PC, uro-oncological guidelines recommend the use of IHC or NGS testing when evaluating mutations in MMR genes or MSI. However, an NGS test validated for PC is preferable for MSI detection. The FoundationOne CDx NGS assay is the only test approved by the FDA to identify tumors with high levels of TMB and the cutoff value ≥10 mutations per megabase (mut/Mb) was associated with sensitivity to pembrolizumab. However, the optimal cutoff value to define high TMB in PC needs further validation.

### 4.3. Androgen Receptor Splice Variant 7 (AR-V7) 

This is a predictive biomarker for decreased response/resistance to ARSI (abiraterone-ABR/enzalutamide-ENZA) and for response to taxane agents not ARSI (AR-V7 positive patients are less likely to respond to ABR/ENZA while AR-V7 status does not seem to affect the response to taxane agents; also, a prognostic biomarker since it is associated with advanced disease, worse outcome, and poor survival).

AR-V7 is a truncated form of AR that lacks the ligand binding domain for androgen and arises from aberrant mRNA splicing of AR exons 1–3, loss of exons 4–8, and inclusion of cryptic exon 3 into the transcribed AR gene. AR-V7 translocates to the nucleus and is active (drives cell growth) in the absence of androgens. AR-V7 prevalence is low before treatment therefore has little use in ADT treatment naive setting but increases post-ARSI therapy (following progression on ADT; up to 75% post-ABR/ENZA) [112]. It can be detected and measured by RT-PCR or IHC (detects AR-V7 at nuclear expression protein level) in circulating tumor cells (CTC) or whole blood using liquid-biopsy tests (e.g., AdnaTest AR-V7 assay from Qiagen or Epic Sciences ArV7 test) or in tumor tissue-biopsies. 

Several recent scientific reports concluded that AR-V7 expression has a prognostic and predictive role (for decreased response or resistance to ARSI) in mCRPC patients, particularly in those previously exposed to novel AR-targeting agents. Uro-oncological experts from NCCN consider that AR-V7 testing can guide the selection of therapy in post-ABR/ENZA mCRPC patients. 

In 2014, Antonarakis and colleagues first reported on the role of AR-V7 status detected in CTCs by AdnaTest in prediction treatment response in mCRPC patients starting ABR or ENZA (prior chemotherapy or alternative ARSI was permitted) [113]. No PSA response was observed in the AR-V7-positive patients. Furthermore, AR-V7 positive patients had shorter PSA progression-free survival (1.4 months vs. 6.0 months, *p* < 0.001; and 1.3 months vs. not reached, *p* < 0.001 in ENZA- and ABR-cohort of patients, respectively), clinical or radiographic progression-free survival (*p* < 0.001) and overall survival (median OS 5.5 months vs. not reached, *p* = 0.002; and 10.6 months vs. not reached, *p* = 0.006). Multivariate analysis adjusted for expression of full-length AR mRNA showed that AR-V7 detection was a significant predictor of therapeutic response. It was reported also that 6 patients (out of 42) AR-V7 negative at baseline converted to AR-V7 positive. Finally, the authors concluded that mCRPC AR-V7-positive patients may be resistant to ABR or ENZA. However, this study had several limitations: a small number of patients (31 patients enrolled in each cohort), no CTCs counting (therefore it is unclear whether the poor response of AR-V7 patients was related to AR-V7 status or high CTCs), no data about CTC negative patients. As a response to these limitations, Antonarakis et al. provided in 2017 more data on the importance of detection of CTC (positive/negative) and AR-V7 status, separately for first and second-line ARSI [114]. In this prospective single-institution study which enrolled 202 mCRPC patients, PSA response to novel hormonal therapeutical agents was observed in 75.5% of CTCs negative patients, 52.2% of CTCs positive and AR-V7 negative patients, and 13.9% of those with CTCs positive and AR-V7 positive patients. Furthermore, CTC-positive/AR-V7-positive patients had more aggressive forms of the disease. Analysis of all outcomes (PSA response, PSA-PFS, PFS, OS) for the overall cohort and separately for the first and second-line ARSI showed that CTC+/AR-V7+ patients had the worst while CTC negative patients had the best outcome. In the multivariable model, biomarker status remained a significant predictor for all outcomes. Again, the authors reported the conversion of CTC negative to CTC positive/AR-V7 positive or AR-7 negative and of CTC positive/AR-V7 negative samples in CTC negative or CTC positive/AR-V7 positive. None of CTC positive AR-V7 positive patients converted to CTC negative. 

Moreover, Armstrong et al. performed a multicenter, prospective study (PROPHECY trial) [115] to validate the prognostic significance of baseline CTC AR-V7 for the clinical utility of ARSI [115]. A total of 118 patients with high-risk mCRPC (defined by 2 or more high-risk prognostic factors: hemoglobin < 12.0 g/dL; alkaline phosphatase > ULN; LDH > ULN; prior therapy with ENZA, ABR, or orteronel; visceral metastasis; pain requiring opioid use; PSA doubling time on most recent therapy < 3 months; radiographic progression based on new lesion(s) in bone, soft tissue, or visceral metastases) starting therapy with ABR or ENZA were enrolled in this trial and detection of CTC AR-V7 was measured by either AdnaTest (AR-V7 mRNA) or Epic sciences (AR-V7 nuclear localization). Although the detection rate differed (82% concordance) and confirmed treatment outcomes (PSA or objective response) were observed also (albeit rare) in AR-V7 positive patients (0% in AR-V7 Epic; 11% PSA response and 6% objective response in AR-V7 AdnaTest), multivariate analysis demonstrated that AR-V7 detection by both tests was independently associated with shorter PFS and OS [115]. 

Sharp et al. performed a clinical utility study addressing the reproducibility of AdnaTest and the association of CTC AR-V7 status with clinical characteristics, CellSearch CTC counts (the only FDA-approved platform for CTC count), AR-V7 protein expression in tissue biopsy using IHC and OS. Interestingly, results of this study showed numerous discrepancies like false negative results (63% of CTC negative and 62% of CTC+/AR-V7- patients had positive AR-V7 protein in tissue biopsies), patients CTC+/AR-V7+ without AR-V7 nuclear protein detection (7% of cases) or detection of CTC counts by CellSearch in Adna CTC negative samples [112]. While the authors suggested that high CTC in AR-V7 + might be a surrogate biomarker in mCRPC, strong scientific evidence is required before implementation of CTC/AR-V7 status in routine clinical practice particularly in treatment decision-making for mCRPC patients. In addition, even though detection of AR-V7 was associated with worse OS and decreased response to ARSI in numerous articles, other studies reported a clinical benefit in a small group of patients regardless of AR-V7 status [115,116,117]. One explanation could be the limitations of detection assays and the possibility that detection of CTC/AR-V7 or nuclear localization of AR-V7 might be better predictors of resistance to ARSI and treatment selection.

Recently, researchers tried to evaluate the effectiveness of taxane-based agents in mCRPC expressing AR-V7 since AR-V7 increase signaling and AR amplification independent of microtubule activity and this sustained activity compromises the taxane-induced effect on the AR translocation into the nucleus. Thus, AR-V7 status might be involved also in the relative resistance mechanism and decreased response to the antimitotic activity of taxane agents. While several papers demonstrated that AR-V7 positive patients starting taxane agents like docetaxel or cabazitaxel (first or second-line setting) had worse outcomes (shorter PFS, PSAPFS, PSARR, OS) compared with AR-V7 negative patients and taxane-agents might be preferred over ARSI in AR-V7 positive patients, experts do not consider AR-V7 status as a predictor of taxane-agents resistance since treatment response was observed irrespective of AR-V7 status [118,119,120,121,122]. Moreover, several limitations were noticed and therefore the evidence supporting the AR-V7 positivity as a predictor of taxane-agents resistance needs further improvements (larger prospective trials, validated assays, other endpoints, comparative effectiveness analysis).

To conclude, experts recommend AR-V7 testing in treatment decision-making for mCRPC progressing after ARSI, particularly in aggressive forms of disease defined by PROPHENCY criteria. In this setting, AR-V7 positive patients will benefit from taxane (more than switching to other ARSI) while AR-V7 negative patients are eligible for both therapies. In addition, based on the results of the randomized, open-label, multicenter CARD trial, cabazitaxel should be the preferred option in patients who had received prior docetaxel and either ABR or ENZA, irrespective of AR-V7 status (rPFS 8.0 vs. 3.7 months) [123]. Another use of AR-V7 testing may be in patients unfit for chemotherapy to inform them that considering a second treatment with another ARSI may be less effective or even ineffective.

### 4.4. TP53, RB1 and PTEN Defects

Recently, molecular characterization of tumor tissues has demonstrated that defects in TP53 (tumor protein 53), RB1 (retinoblastoma 1), and PTEN (phosphatase and tensin homolog) are associated with PC progression and aggressiveness. Moreover, these biomarkers were associated with poor clinical outcomes and therapeutic resistance.

TP53 gene regulates the nuclear transcriptional process mainly in response to DNA-damaging agents. Mutations in TP53 abrogate cell cycle G1 checkpoint and promote genomic instability. It is most frequently detected in advanced PC (60–90%) and is considered a hallmark of an aggressive variant of the disease. However, the presence of TB53 mutations in the RP specimens was associated with distant metastases, aggressive behavior, and androgen resistance.

Although the role of TP53 mutation in prostate cancer is controversial, several articles demonstrated its association with shorter PFS and OS in patients receiving ABR or ENZA [124]. Furthermore, other studies showed that the presence of TP53 mutations affected the response to docetaxel or olaparib (PARP inhibitor) [94,125]. How TP53 alterations confer resistance to ARSI, docetaxel or olaparib is currently unknown. Interestingly, androgen independence and lineage plasticity (concept of the mechanism of resistance defined by the ability of cancer cells exposed to anticancer agents to modify their pathway of cellular differentiation process and less dependency on the targeted oncological driver) were suggested to account for this therapeutic resistance [126,127].

RB1 (a regulator of cell cycle G0/G1 phase) and PTEN are other most frequently altered genes in advanced prostate cancer. Some studies reported a prognostic role of RB1 in progression and poor clinical outcome in mCRPC patients [128,129]. However, its significance remains unclear.

PTEN loss is a genetic alteration event that occurs since the early stage of PC and is detected more frequently in metastatic PC (40–60% vs. 5–27% in localized PC) [130,131]. It is measured by quantification of protein expression level with IHC (gold standard technique) (10% of cancer cells presenting positive staining was considered the cutoff value in some studies while 50% or more of the specimen’s tumor area having no detectable PTEN in others) [132,133]. PTEN loss was associated with aggressive behavior of PC (worse prognosis) and demonstrated a prognostic role in low-risk localized PC (endpoint: upgrading the biopsy grade group to higher grade on RP; may identify unsuitable low-risk patients for AS; insufficient data to be recommended in clinical practice) or RP-treated patients (endpoint: biochemical recurrence; conflicting data) and a possible predictive role in mCRPC (resistance to therapy, particularly to abiraterone) [132].

PTEN loss causes overactivation of the PI3K/AKT pathway and, consequently impacts the AR signaling pathway thus providing a rationale for combined therapy (AR-targeted therapies and PI3K/AKT inhibitors). Sweeney et al. performed a randomized trial to investigate the efficacy of dual pathway inhibition using a PI3K/AKT inhibitor and an ARSI in mCRPC patients with PTEN loss. The authors showed that ipatasertib (orally 400 mg daily) plus abiraterone (orally 1000 mg daily plus prednisolone 5 mg twice daily) increased radiographic progression-free survival in cases with PTEN loss (18.5 months vs. 16.5 months in placebo plus abiraterone group) [133]. Nevertheless, studies are showing that PTEN status did not confer resistance to docetaxel [134]. On the other hand, Corn et al. showed that carboplatin added to cabazitaxel (taxane agent) improved median progression-free survival and median overall survival in mCRPC patients with aggressive variants of PC including PTEN loss [135].

Therefore, NCCN guidelines v1.2022 state that cabazitaxel plus carboplatin should be considered for prostate cancer patients with unfavorable genomics defined by defects in at least 2 of TP53, Rb1, and PTEN.

### 4.5. Circulating Tumor Cells (CTC)

CTS are prognostic (for OS) and a predictive biomarker for therapeutic response in mCRPC

CTC have been investigated as liquid biopsy biomarkers in PC, particularly in patients with mCRPC. In clinical practice, CTC can be detected in the blood of patients with mCRPC using the FDA-approved CellSearch platform (Janssen Diagnostics, Raritan, NJ, USA). Detection of CTC was correlated with OS in numerous studies and a baseline CTC count > 5 per 7.5 mL of blood was associated with decreased OS. Median OS was significantly shorter in mCRPC patients with baseline CTC count > 5 CTC per 7.5 mL of blood (compared with those with < 5 CTC per 7.5 mL of blood) who received docetaxel (11.5 months versus 21.7 months), ABR postchemotherapy (10.9 months versus 22.1 months) or ENZA postchemotherapy (13.6 months versus not reached) [136,137,138]. Moreover, changes in CTC counts from baseline during treatment (favorable to unfavorable CTC counts or vice versa) were correlated with OS even stronger than PSA decrement algorithms at all time points. A phase 3 prospective clinical trial comparing docetaxel versus docetaxel with atrasentan showed that rising CTC count from baseline to day 21 significantly shortened/worsened OS (HR 2.55) [139]. In another study median OS was improved for mCRPC patients with unfavorable baseline CTC count who converted to favorable CTC count after treatment (6.8 months to 21.3 months) [136].

In addition, changes in CTC count may be a promising predictive biomarker of treatment response. A metanalysis of five prospective randomized phase 3 clinical trials (NCT00638690—Abiraterone Acetate in Castration-Resistant Prostate Cancer Previously Treated With Docetaxel-Based Chemotherapy, NCT00974311—Safety and Efficacy Study of MDV3100 in Patients With Castration-Resistant Prostate Cancer Who Have Been Previously Treated With Docetaxel-based Chemotherapy (AFFIRM), NCT01193257—Study Comparing Orteronel Plus Prednisone in Participants With Metastatic Castration-Resistant Prostate Cancer, NCT01193244—Study Comparing Orteronel Plus Prednisone in Participants With Chemotherapy-Naive Metastatic Castration-Resistant Prostate Cancer, and NCT01605227—Study of Cabozantinib (XL184) Versus Prednisone in Men With Metastatic Castration-resistant Prostate Cancer Previously Treated With Docetaxel and Abiraterone or MDV3100 (COMET-1)) comparing week 13 CTC count and changes in PSA level as treatment response end points demonstrated that CTC0 (conversion from baseline CTC count ≥ 1 to 0 or not detectable CTC count at week 13 posttreatment) and CTC conversion from baseline CTC counts ≥ 5 to ≤ 4 at week 13 were superior treatment response endpoints to the widely used PSA algorithms such as 30%, 50%, and 70% decrease in PSA level and concluded that CTC0 should be implemented in clinical trials as a reliable predictive biomarker of early treatment response efficacy [140].

While the clinical utility of CTC is very promising it still needs further validation and improvement.

### 4.6. Cell-Free DNA (cf-DNA)

Cf-DNA is a prognostic and predictive biomarker for OS and therapeutic response in mCRPC (taxane therapy). Cf-DNA is another promising liquid biomarker for advanced PC (more stable than CTC). Cf-DNAs are small fragments of nucleic acid that are released from lysed and apoptotic PC cells. The presence of cf-DNA in the plasma was shown to improve the diagnosis of PC in patients with increased serum PSA and was associated with biochemical recurrence after RP [141,142]. In mCRPC, several studies demonstrated the clinical utility of cf-DNA as a prognostic biomarker for OS and to identify patients who may respond to taxane agents. In a retrospective study, increased cf-DNA concentration (optimal cutoff value 55.03 ng/mL) was associated with poor PSA response and OS (17 months in patients with cf-DNA concentration ≥ 55.03 ng/mL and 31.5 months in those with <55.03 ng/mL) [143]. Additional research studies showed that changes in cf-DNA concentration during treatment with docetaxel or cabazitaxel were associated with rPFS and OS, supporting the predictive value of this biomarker for response to taxane-based chemotherapy [144]. Of note, patients with a favorable treatment response had a decline in cfDNA concentration during the first four cycles of taxane therapy.

Like CTC, continuing scientific efforts are necessary to certify the clinical utility of cf-DNA in the management of PC.

### 4.7. Periprostatic Adipose Tissue Releasing Factors

Periprostatic adipose tissue (PPAT)-releasing factors have been associated with PC aggressiveness and response to therapeutical agents. Thickness of PPAT, PPAT/subcutaneous adipose tissue ratio, or multiple releasing factors were evaluated for prognosis or predictive significance in metastatic PC. The level of circulating cytokines (IL6, IL8, IL23), and expression of PD 1 receptor or CD38 on immune cells are a few examples of promising immunological biomarkers studied in the context of the aggressive behavior of PC [145]. Antonietta Liotti et al. explored the role of factors released by PPAT on the mechanism of resistance to docetaxel in mCRPC [146]. The authors found that upregulation of IGF-1 and increased BCL-XL, BCL-2, and TUBB2B protein expression are involved in docetaxel resistance. In addition, AG1024 an IGF1 receptor inhibitor reverses the response to docetaxel suggesting a potential strategy to overcome docetaxel resistance in metastatic PC [146].

## 5. Conclusions

In this review, we presented with respect to the latest scientific evidence some of the most robust and reliable prognostic and predictive biomarkers that may help clinicians, particularly in difficult-to-manage scenarios of PC, a disease characterized by heterogeneity in clinical and biological (including genomic) behavior. A summary of these biomarkers and their potential clinical utility are illustrated in Table 1, Table 2, Table 3 and Table 4. While currently, serum PSA remains the only, universally accepted and most frequently used biomarker in urological practice worldwide for early diagnosis (screening), definitive diagnosis (prostate biopsy), prognosis, and any therapeutic decisions of PC, experts recommend its use together with other tools such as biomarkers, RC or new imaging techniques to identify aggressive forms of the disease. Since uro-oncological guidelines are improved every year with new treatment options and therapeutic agents it is expected that, in the near future, biomarkers will be incorporated into clinician’s standard of care.

## Figures and Tables

**Table 1:** Biomarkers recommended by experts to improve the probability of detection of csPC.

Urooncological Guidelines	Initial Biopsy	Repeat Biopsy
NCCN v2.2023 prostate cancer early detection [7]	%fPSA, PHI,4K, Select MDX, EXO Dx (EPI), MyProstateScore(MPS), IsoPSA	%fPSA, PHI,4K, EXO Dx (EPI), PCA3, Confirm MDX, MPS, IsoPSA
EAU-EANM-ESTRO-ESUR-SIOG Guidelines on Prostate Cancer 2023 [6]	PHI,4K, PCA3, Select MDX, MiPS, EXO Dx (EPI), IsoPSA	PHI,4K, PCA3, Select MDX, Confirm MDX

**Table 2:** Summary of potential clinical utility of biomarkers recommended by experts to improve the detection of csPC.

Assay (Company)	Biomarkers Used	Output	Result Interpretation	Potential Clinical Use
PHI (Beckman Coulter Inc., Brea, CA, USA)	free PSA, [-2] proPSA, total PSA	PHI score (range 0–100)	- cutoff = 27 (prior mpMRI negative) - biopsy recommended at PHI score ≥ 36 - a higher score is associated with a greater risk of detecting PC, higher pathological GS, and stage	- Screening/AS to improve biopsy decisions in patients with suspicious PSA levels and normal DRE - Stronger predictor in combination with ERSPC RC, MRI, or 4K score
4K (OPKO Lab, Nashville, TN, USA)	total PSA, free PSA, intact PSA and human kallikrein protein 2	4k score (range 0–100)	- prediction of csPC in patients with suspicious PSA level or DRE - Low risk 1–7.5 - High risk ≥ 20 - higher risk is associated with a higher probability of detecting csPC, aggressive pathology, or progression to metastasis or death	- screening/AS - prediction of developing PC-metastasis - better predictive performance in combination with RPCRC, MRI
PCA3 (ProgensaTM PCA3 assay; Gen-Probe, San Diego, CA, USA)	mRNA PCA3, mRNA PSA	PCA3 score (range 0–>100)	- Cutoff stratification (<20, 20–60, >60) for prediction of positive biopsy - increasing PCA3 scores were associated with a high probability of detecting any PC and HGPC	- rebiopsy decision-making in patients with suspicious PSA levels and normal DRE - better predictive performance in combination with RC (PCPTRC, ERSPC RC4) and among patients with low suspicion scores on MRI at PCA3 score > 35
Michigan prostate (MiPS, Mlabs, Ann Arbor, MI, USA)	serum PSA and two urinary biomarkers (PCA3 and TMPRSS2-ERG)	Mi Score (range 0–100%).	- Mi score of ≤10 could exclude GG cancer ≥ 2 - the higher the score, the greater the risk of aggressive cancer	- prediction of csPC in patients with suspicious PSA levels - better predictive performance MiPS with PSA, PCA3, or RC (PCPTRC).
Select MDX (MDxHealth, Inc., Irvine, CA, USA)	mRNA of HOXC6 (proliferation gene) and DLX1 genes (progression gene)	Risk score (−6 to 6) Cutoff −2.8	- Positive test—a risk score ≥ −2.8 - the higher the score, the greater the risk of aggressive cancer.	- prediction of csPC in patients with suspicious PSA level ± abnormal DRE to be considered in combination with MRI-based strategy (PSA > 3 ng/mL and PIRADS score 4–5 or PIRADS score 1–3 followed by a positive Select MDX)
ExoDx Prostate (INTELLISCORE) (EPI; Exosome Diagnostics, Cambridge, MA, USA)	3 urinary exosomal RNAs (ERG and PCA3 normalized to SPDEF)	Risk score (0–100)	- Cutoff 15.6 - high-risk of PC (GS > 7) > 15.6	- prediction of csPC among patients with suspicious serum PSA level or DRE - better combined with standard-of-care clinical variables (PSA level, age, race, family history)
Confirm MDX assay (MDxHealth, Inc., Irvine, CA, USA)	GSTP1, APC and RASSF1	DNA methylation	- DNA methylation positive- consider rebiopsy	- prediction of PC in repeat biopsy settings - data regarding its clinical utility in predicting csPC are expected.

**Table 3:** Summary of potential clinical utility of biomarkers recommended by experts to be considered in the management of localized PC.

Assay (Company)	Biomarkers/Samples Used	Output	Result Interpretation	Potential Clinical Use
Decipher (GenomeDx, San Diego, CA, USA)	RNA expression of 22 genes (tissue from biopsy or RP specimen)	Decipher score (range 0–1)	- low risk (range 0–0.45); - intermediate risk (0.45–0.6) - high risk (0.6–1)	- Prediction of adverse pathology at RP (on biopsy cores) - Prediction of metastasis post-RP and post-RT - Guidance in decision-making post-RP regarding the indication of RT
Oncotype Dx (OncotypeDx GPS, Genomic Health, Redwood, CA, USA)	RNA expression of 17 genes (tissue from biopsy)	Genomic Prostate score (range 0–100)	- the higher the score, the greater the risk of aggressive cancer	- prediction of adverse pathological features on RP specimens or aggressive behavior of clinically localized PC (metastasis, PC death).
Prolaris (Myriad Genetic Laboratories, Salt Lake City, UT, USA)	RNA expression of 46 genes (tissue from biopsy or RP specimen)	cell-cycle progression score (range 0–10)	- the higher the score, the greater the risk of aggressive cancer	- prediction of aggressiveness post-biopsy or post-RP
ProMark (Metamark, Cambridge, MA, USA)	expression of 8 proteins (tissue from biopsy)	score (range 0–1)	- the higher the score, the greater the risk of aggressive cancer	- prediction of adverse pathology on RP specimens

**Table 4:** Summary of potential clinical utility of biomarkers recommended by experts to be considered in the management of mCRPC.

Biomarker/Sample	Recommended/Potential Clinical Use
HRR-gene mutations (blood, saliva, or tumor tissue-metastatic sites or archival)	PARP inhibitors Olaparib following enzalutamide or abiraterone for a specific panel of HRR-gene mutations, irrespective of prior taxane-based therapy Rucaparib following enzalutamide or abiraterone only for BRCA1/2 mutations, irrespective of prior taxane-based therapy Niraparib for BRCA1/2 mutations, irrespective of prior therapy Talazoparib is better for BRCA1/2 mutations (not indicated following abiraterone, enzalutamide, or docetaxel)
	Taxane-based therapy (Docetaxel, Cabazitaxel)
DNA mismatch repair gene mutations (dMMR)- (MLH1, MSH2, MSH6, and PMS2) or high level of microsatellite instability (MSI-H) (blood, tumor tissue)	PD-1 immune checkpoint inhibitors (pembrolizumab) following at least one line of systemic therapy AND no satisfactory available alternative treatment options (FDA approval does not explicitly include PC)
High levels of tumor mutational burden (TMB-H) (blood, tumor tissue)	PD-1 immune checkpoint inhibitors (pembrolizumab) following at least one line of systemic therapy AND no satisfactory available alternative treatment options (FDA approval does not explicitly include PC)
Androgen receptor splice variant 7 (AR-V7) (blood)	predictive biomarker for decreased response/resistance to ARSI (abiraterone-ABR/enzalutamide-ENZA) and for response to taxane agents, not ARSI (AR-V7 positive patients are less likely to respond to ABR/ENZA while AR-V7 status does not seem to affect the response to taxane agents) prognostic biomarker associated with advanced disease, worse outcome, and poor survival
TP53, RB1, and PTEN defects (tumor)	biomarkers associated with poor clinical outcome and therapeutic resistance
Circulating tumor cells (CTC) (blood)	prognostic (for OS) and predictive biomarker for therapeutic
Cell-free DNA (cf-DNA) (blood)	prognostic and predictive biomarker for OS and therapeutic response (taxane-therapy)
